# Assessment of articular cartilage of ankle joint in stable and unstable unilateral weber type-B/SER-type ankle fractures shortly after trauma using T2 relaxation time

**DOI:** 10.1177/20584601231202033

**Published:** 2023-09-29

**Authors:** Sami Lehtovirta, Victor Casula, Marianne Haapea, Simo Nortunen, Sannamari Lepojärvi, Harri Pakarinen, Miika T. Nieminen, Eveliina Lammentausta, Jaakko Niinimäki

**Affiliations:** 1 Research Unit of Health Sciences and Technology, 6370 University of Oulu, Oulu, Finland; 2 Medical Research Center, Oulu University Hospital, 6370 University of Oulu, Oulu, Finland; 3 Department of Diagnostic Radiology, 60664 Oulu University Hospital, Oulu, Finland; 4 Pihlajalinna Hospital, Oulu, Finland

**Keywords:** magnetic resonance imaging, cartilage, ankle, trauma, osteoarthritis

## Abstract

**Background:**

Early detection of post-traumatic cartilage damage in the ankle joint in magnetic resonance images can be difficult due to disturbances to structures usually appearing over time.

**Purpose:**

To study the articular cartilage of unilateral Weber type-B/SER-type ankle fractures shortly post-trauma using T2 relaxation time.

**Material and Methods:**

Fifty one fractured ankles were gathered from consecutively screened patients, compiled initially for RCT studies, and treated at Oulu University Hospital and classified as stable (*n* = 28) and unstable fractures (*n* = 23) based on external-rotation stress test: medial clear space of ≥5 mm was interpreted as unstable. A control group of healthy young individuals (*n* = 19) was also gathered. All ankles were imaged on average 9 (range: 1 to 25) days after injury on a 3.0T MRI unit for T2 relaxation time assessment, and the cartilage was divided into sub-regions for comparison.

**Results:**

Control group displayed significantly higher T2 values in tibial cartilage compared to stable (six out of nine regions, *p*-values = .003–.043) and unstable (six out of nine regions, *p*-values = .001–.037) ankle fractures. No differences were detected in talar cartilage. Also, no differences were observed between stable and unstable fractures in tibial or talar cartilage.

**Conclusions:**

Lower T2 relaxation times of tibial cartilage in fractured ankles suggest intact extra cellular matrix (ECM) of the cartilage. Severity of the ankle fracture, measured by ankle stability, does not seem to increase ECM degradation immediately after trauma.

## Introduction

Over 3% of individuals older than 50 years suffer from osteoarthritis (OA) of the ankle joint.^1^ Although the ankle is exposed to higher contact pressures compared to the knee or hip, primary OA of the talocrural ankle joint is rather rare compared to the primary OA of the knee and hip joints.^2^ This is speculated to be due to the difference in joint functions: the ankle functions as a rolling high-congruent joint, whereas the knee has a combination of sliding, rotation, and rolling capabilities.^3^

Multiple studies have demonstrated that the most common etiology of the ankle OA is post-traumatic: up to 80% is estimated to result from an injury.^2,4,5^ The major risk factor for developing ankle OA is a previous ankle fracture and the severity of the initial injury is thought to be the most important prognostic feature.^4^ Ankle OA can result from incongruence and instability of the ankle mortise due to the trauma or suboptimal treatment.^6,7^

The Weber type-B ankle fracture consists of fractured fibula at the level of the ankle joint that extends proximally^8^ and is most often due to supination-external rotation (SER) mechanism of injury,^9–12^ with incidence rates reported to be up to more than 75% of all ankle fractures.^11,13^ However, this most common fracture type is a heterogeneous group of injuries, as a fractured ankle (ankle mortise) can be stable or unstable depending on the possible medial side injury.^10–12^ A common approach in determining the stability of unilateral ankle fractures is the external-rotation stress test, which is the most reliable means to assess the stability of the ankle mortise.^12,14–16^ In the test, the injured ankle is manually rotated externally and radiographed. The test is usually deemed positive, indicating unstable fracture, if the space between the medial malleolus and talar dome (medial clear space, MCS) is ≥5 mm (mm).^11,12,14,15^ A positive stress test indicates a significant tear and instability of the deep deltoid ligament.^11,12,14,15,17^

Radiological evaluation of post-traumatic cartilage damage within few weeks after injury can be difficult due to visual disturbances to joint structures usually appearing over time in images. Therefore, it can be challenging to distinguish whether the cartilage damage was caused by the actual trauma or developed over time due to altered limb mechanics or long-term inflammation, for example.^18^ However, quantitative magnetic resonance imaging (qMRI) methods have been shown to provide good results in detecting early OA-related changes non-invasively in articular cartilage.^19–22^

T2 relaxation time is an established qMRI parameter, which has been proven effective in evaluation of articular cartilage structure^19,22–24^ and mechanical properties.^21^ T2 relaxation is affected by the collagen and water content, as well as by collagen fibril network orientation and integrity.^20,23,25^ Elevated T2 relaxation time values are associated with degenerative changes in the extracellular matrix (ECM) of articular cartilage.^26,27^

We have previously reported lacking value of visual MRI assessment when evaluating the stability of ankle mortise in SER-type ankle fractures.^16^ However, no previous studies have assessed cartilage damage in humans shortly after trauma using qMRI. Therefore, we set out to evaluate ankle articular cartilage post-trauma in patients reported previously by Nortunen et al.^16^ Our aim was to study the articular cartilage of unilateral SER-type ankle fractures shortly after injury using T2 relaxation time.

## Material and methods

### Subjects

The study group consisted of skeletally mature ankle fracture patients (16 years of age or older) treated at Oulu University Hospital between March 2012 and April 2013. Consecutive patients with an isolated Weber B/SER-type lateral malleolar fracture and congruent ankle mortise (MCS <4 mm and ≤1 mm wider than the superior clear space) were initially enrolled for pragmatic randomized controlled trials (RCT).^28,29^ All patients gave written informed consent. A total of 87 study participants were gathered in the previous study^16^ of which 26 could not be included due to variety of reasons reported previously^16^; five were omitted due to severe metal artifacts, three due to subpar quality of MR images, and two because T2 maps were not acquired. The metal artifacts caused severe anatomical distortion in the MR images and led to inaccurate T2 maps. Additionally, the three patients with bad quality MR images exhibited increased amounts of noise in the images, resulting in visible degradation of the anatomical details in T2-weighted images. Image quality was qualitatively assessed by a radiologist (S.Lepojärvi) and MR physicists (V.C, E.L). The final study group contained 51 subjects (23 female and 28 male; mean age 45 years, standard deviation (SD) 18 years; age range 16 to 82 years). The study group was split into two RCTs^28,29^ based on the external-rotation stress test: stable fractures (*n* = 28; 12 female, 16 male; mean age 42 years, SD 18 years; mean BMI 26.7 kg/m^2^, SD 4.9 kg/m^2^) and unstable fractures (*n* = 23; 11 female, 12 male; mean age 48 years, SD 19 years; mean BMI 26.9 kg/m^2^, SD 4.2 kg/m^2^). Demographics are presented in Table 1.

**Table 1.** Demographic characteristics of the control group, and stable and unstable fracture groups. Values for age and body mass index (BMI) are presented as means with standard deviations (SD) and ranges (mean ± SD (minimum – maximum)).

	Control	Stable	Unstable	Statistical significance, *p*-values		
				Control vs. Stable	Control vs. Unstable	Stable vs. Unstable
N	19 (7 female, 12 male)	28 (12 female, 16 male)	23 (11 female, 12 male)	0.77^b^	0.54^b^	0.78^b^
Age (years)	22 ± 2 (19 – 25)	42 ± 18 (18 – 81)	48 ± 19 (16 – 82)	<0.001^a^	<0.001^a^	0.53^a^
BMI (kg/m^2^)	22.1 ± 1.9 (18.8 – 25.4)	26.7 ± 4.9 (20.3 – 39.0)	26.9 ± 4.2 (21.3 – 39.6)	<0.001^a^	<0.001^a^	0.97^a^

N: Number of participants; SD: standard deviation; BMI: body mass index

^a^Games-Howell post hoc test

^b^Pearson Chi-Square test, difference for gender distribution.

A control group of healthy young medical students was gathered for our conducted qMRI study. The recruitment of volunteers was conducted via email among medical students. A detailed explanation of the study and its objectives was included in the email, and by replying the volunteers stated their consent for study participation. The group consisted of 23 participants (11 female, 12 male). However, four females had their T2 images lost from the archives, resulting in total of 19 participants (7 female, 12 male) aged from 19 to 25 years (mean 22 years, SD 2 years) with a BMI of 18.8–25.4 kg/m^2^ (mean 22.1 kg/m^2^, SD 1.9 kg/m^2^). The participants did not have any previous injuries of ankle or any other condition, which would affect lower limb biomechanics. This group was chosen to represent healthy adult cartilage with minimal age-related cartilage deterioration.

### External-rotation stress test

External-rotation stress test was performed to determine the width of MCS for each patient. Detailed description of the test can be found in the previous study.^16^ MCS of 5 mm was decided as a cut-off value, due to previous studies suggesting a MCS width of ≥5 mm to indicate unstable ankle mortise due to significant tear of the deep deltoid ligament and indicating operative treatment method.^11,12,14,15,17,30,31^

### Magnetic resonance image analysis

Each patient went through examination on a 3.0 T MAGNETOM Skyra (Siemens Healthcare, Erlangen, Germany) MRI unit following splint placement of the fractured extremity. The MRI protocol included a multiecho spin-echo sequence for T2 mapping (TR = 1810 ms; TE^5^ = 13.8–69.0 ms; ETL = 5; FOV = 159 × 159 mm^2^; Matrix = 384 × 384; Thickness = 3 mm; Slice gap = .6 mm; number of slices = 22; sagittal plane). The average delay from the time of injury to MRI was 9 ± 6 days (range 1 to 25 days).

**Figure 1.**
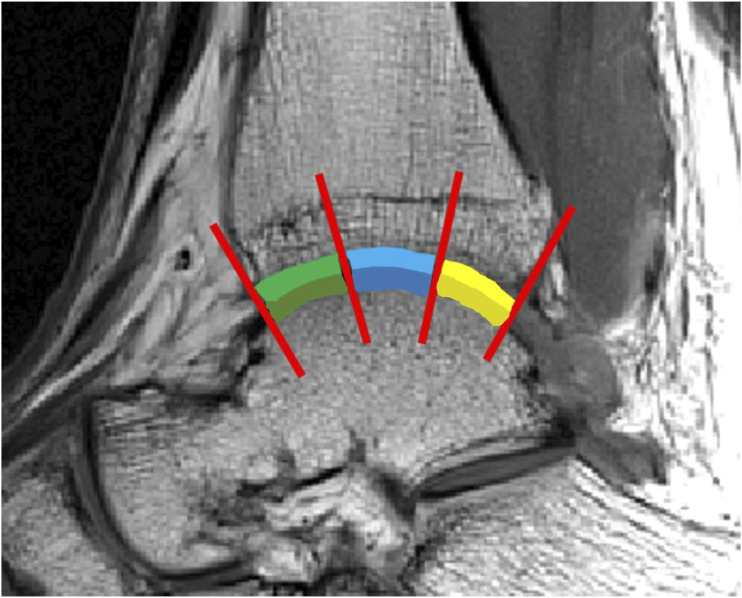
Sagittal T2-weighted image of ankle joint from a 20-year-old male (stable fracture). Each of the studied three regions is indicated by their respective color: green = anterior region, blue = middle region, yellow = posterior region. Lighter and darker color indicate tibial and talar cartilage, respectively.

**Figure 2.**
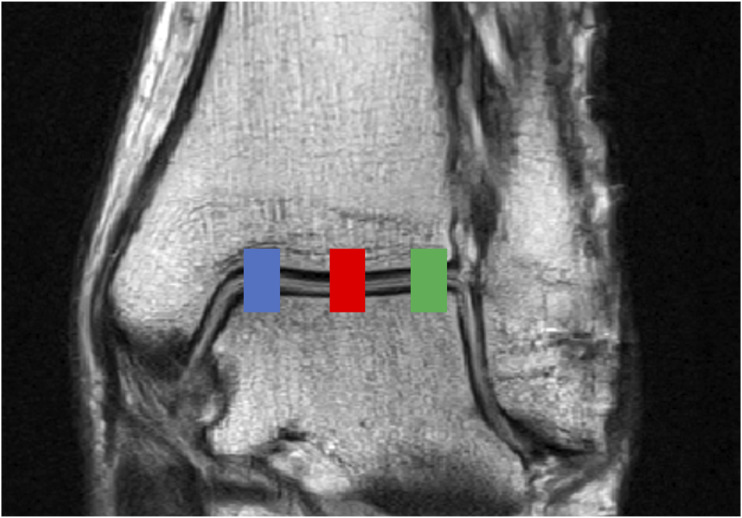
Coronal T2-weighted image of ankle joint from a 43-year-old male for demonstration purposes. Each of the studied three sections is indicated by their respective color: blue = medial section, red = central section, and green = lateral section.

The average T2 relaxation times were obtained from sagittal T2 maps of ankle mortise in three regions (anterior, middle, posterior) (Figure 1) and three different sections (medial, central, lateral) (Figure 2). In-depth description of ankle mortise segmentation and slice selection is provided in the Online Supplemental Material.

### Statistics

T2 relaxation times are presented as means with standard deviations for each region of interest (ROI). Differences between the groups were assessed using analysis of variance (ANOVA) with Bonferroni’s pairwise comparison. The differences of age and BMI between the groups were assessed using ANOVA with Games-Howell post hoc test due to difference of variances. The difference in gender distribution was evaluated by Pearson Chi-Square test. The T2 relaxation time analyses were adjusted for age and BMI due to the difference of these values between the groups (Table 1). A *p*-value <.05 was considered statistically significant. The statistical analyses were conducted using IBM SPSS for Windows, version 27.

## Results

**Figure 3.**
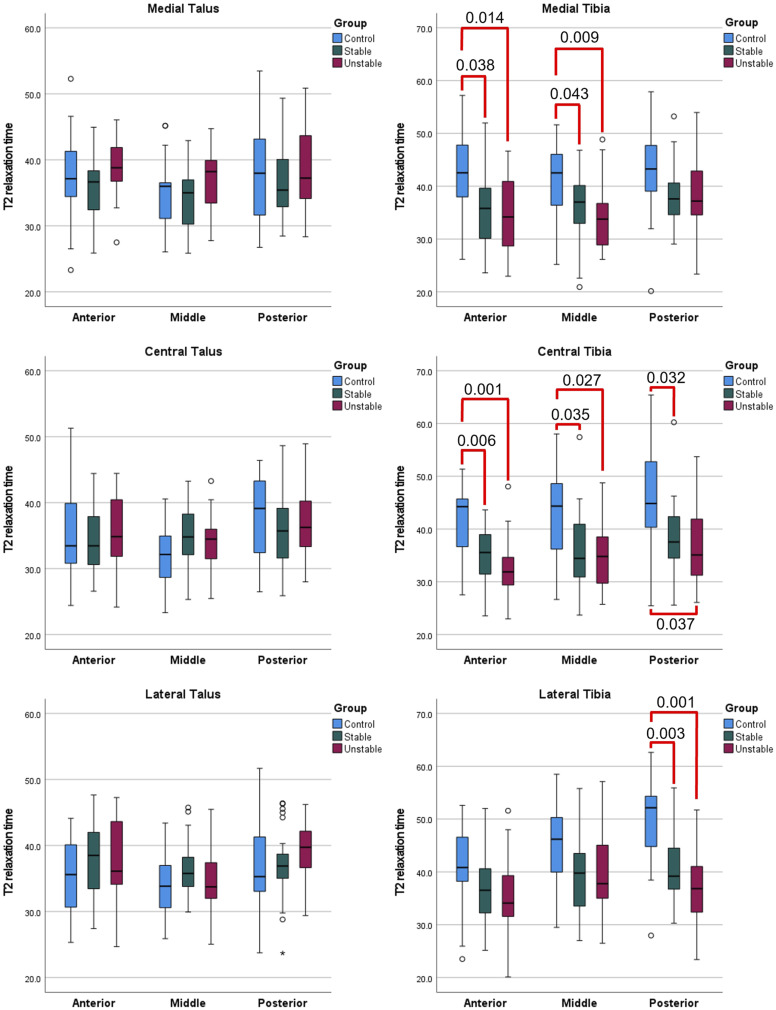
Mean T2 relaxation times presented separately for each region of interest. Statistical differences are displayed with bright red color and respective *p*-values. Studied groups are indicated by their respective color: blue = control group, green = stable fracture group, and dark red = unstable fracture group.

A significant difference in mean T2 relaxation times was detected in six out of nine ROIs in tibial cartilage between the control group and stable fractures, with higher values displayed by the control group (medial anterior and middle regions, *p* = .038 and .043, respectively; central anterior, middle, and posterior regions, *p* = .006, .035, and 0.032, respectively; lateral posterior region, *p* = .003). No differences were observed between control group and stable fractures in talar cartilage (Table 2, Figure 3).

**Table 2.** Mean T2 relaxation times presented with standard deviations (SD) for each region of interest (ROI). Statistical differences between the three groups (control group, stable fracture group, unstable fracture group) indicated by *p*-values for each ROI separately, with a *p*-value of <.05 indicating statistical significance.

	Control		Stable		Unstable		Statistical significance, *p*-values		
	N	Mean (SD)	N	Mean (SD)	N	Mean (SD)	Control vs. Stable	Control vs. Unstable	Stable vs. Unstable
Talus									
Medial									
Anterior	18	37.4 (7.1)	28	35.8 (4.4)	22	38.6 (4.3)	>0.99	>0.99	0.24
Middle	19	34.9 (5.7)	28	34.3 (4.2)	22	37.2 (4.5)	>0.99	>0.99	0.14
Posterior	19	37.9 (7.7)	28	36.6 (5.2)	22	38.4 (6.1)	>0.99	>0.99	>0.99
Central									
Anterior	19	35.0 (7.2)	28	34.2 (4.6)	23	35.5 (5.4)	>0.99	>0.99	>0.99
Middle	19	32.0 (4.7)	28	34.9 (4.7)	23	34.0 (4.2)	>0.99	>0.99	0.98
Posterior	19	37.5 (6.7)	28	35.9 (5.5)	23	37.7 (6.0)	0.94	>0.99	0.93
Lateral									
Anterior	19	35.3 (5.7)	27	37.8 (5.2)	22	37.4 (6.2)	0.57	0.92	>0.99
Middle	19	34.0 (5.0)	27	36.2 (4.0)	22	34.3 (4.2)	>0.99	>0.99	0.25
Posterior	19	36.9 (6.9)	27	37.2 (5.3)	21	38.8 (4.8)	>0.99	>0.99	>0.99
Tibia									
Medial									
Anterior	19	43.0 (7.1)	26	36.3 (8.1)	22	34.6 (6.9)	**0.038**	**0.014**	>0.99
Middle	19	41.0 (7.2)	27	36.2 (6.7)	22	34.1 (6.1)	**0.043**	**0.009**	0.77
Posterior	18	42.1 (8.5)	27	38.3 (5.6)	22	37.9 (7.0)	0.93	0.92	>0.99
Central									
Anterior	19	41.1 (7.4)	27	35.0 (5.1)	22	32.7 (5.9)	**0.006**	**0.001**	0.61
Middle	19	43.1 (9.1)	27	36.2 (7.1)	23	35.1 (6.6)	**0.035**	**0.027**	>0.99
Posterior	18	46.0 (10.7)	27	38.3 (6.8)	20	37.4 (7.9)	**0.032**	**0.037**	>0.99
Lateral									
Anterior	19	40.5 (8.6)	26	36.7 (6.3)	21	35.4 (7.2)	0.21	0.117	>0.99
Middle	19	44.8 (7.8)	26	40.0 (7.4)	21	39.8 (7.9)	0.51	0.95	>0.99
Posterior	15	49.1 (8.6)	21	40.5 (6.3)	13	37.3 (8.0)	**0.003**	**0.001**	0.45

The control group also displayed significantly higher T2 values compared with unstable fractures in six out of nine ROIs in tibial cartilage (medial anterior and middle regions, *p* = .014 and .009, respectively; central anterior, middle, and posterior regions, *p* = .001, .027, and 0.037, respectively; lateral posterior region, *p* = .001). No differences were observed between control group and unstable fractures in talar cartilage (Table 2, Figure 3).

No differences were observed in tibial or talar cartilage between stable and unstable fractures (Table 2, Figure 3).

## Discussion

Our study describes findings of unilateral SER-type ankle fractures’ effect on cartilage ECM within days after trauma. Our results displayed differences in six out of nine ROIs in tibial cartilage when comparing control group and stable fractures, with control group displaying higher T2 values. Furthermore, when comparing control group with unstable fractures, differences were detected in six out of nine ROIs in tibial cartilage, with the control group displaying higher T2 values. In talar cartilage such differences were not detected. The lower T2 values (of tibial cartilage in the fractured ankles) suggest intact cartilage with no severe damage to cartilage ECM.

It is slightly surprising that the control group displays higher T2 values compared to the fracture groups: higher T2 values usually imply more degraded cartilage quality.^26,27^ Moreover, ankle fractures are usually painful and therefore may be not mechanically loaded shortly after injury, which increases the cartilage interstitial water content,^32^ which in turn should increase T2 relaxation time.^23^ There is age difference between the control group and the fractured ankles, and though this is accounted for in the statistical analysis, it may introduce a potential bias. However, the age difference alone cannot explain the higher T2 observed in the young control group as compared to the fractured ankles. Since T2 relaxation time is known to increase with age, the young control group would be expected to display lower T2 values.^27,33^ Previous studies have observed higher T2 values in children,^34^ although this is not the case in our study, as our healthy young control group represent mature cartilage with minimal age-related cartilage deterioration. The exact reason for our findings may be speculated, although it would seem unilateral SER-type ankle fractures do not degrade the cartilage ECM in the immediate short-term.

We may speculate that a possible explanation for the lower T2 relaxation times in fractured ankles could be due to cartilage compression, which could result from a traumatic impact^35^ leading to increased cartilage density and reduced water content, although the cartilage should be able to regain its original state shortly after.^32^ Lower T2 values have also been associated with cartilage scarring,^24^ although we suspect this was not the case in our study, as imaging was performed shortly after injury. Previous research has also demonstrated that OA subjects may display lower T2 values in some cartilage lesions as compared to normal cartilage in healthy controls. However, the etiology behind these types of findings is not yet understood properly.^36^ We may still hypothesize whether these types of results could explain our findings as well, with fractured ankles actually displaying more compromised cartilage.

Another speculative explanation for our findings could be due to the resolution of the images combined with physiologically thin ankle cartilage: tibial and talar cartilage were each around three to four pixels thick in the sagittal T2 slices. High intensity pixels located between the cartilage surfaces are subject to contain synovial fluid, which increases the T2 relaxation time.^23^ These pixels were omitted from the analysis if the T2 was higher than 90 ms (as described in the Online Supplemental Material), as this highly suggests the inclusion of synovial fluid rather than plain cartilage. These high intensity pixels were more apparent in the fractured ankles, suggesting more synovial fluid interfering with the analysis in fractured ankles compared to the control group. The image resolution may not be high enough to separate synovial fluid from cartilage surface which is known to present physiologically higher T2 values compared to deep segments of cartilage.^37^ The exclusion of hyperintense pixels close to articular surfaces, while reducing the inclusion of synovial fluid in the analysis, may have resulted in the exclusion of degenerated tibial cartilage with higher T2 relaxation times in the fractured ankles. Nevertheless, we believe this alone would not be enough to display such significant differences between the groups. Also, the cartilage was segmented twice because of this to ensure utmost consistency of the segmentation.

There are limited data when it comes to evaluating cartilage damage few weeks after ankle fracture. A previous study by Tochigi et al.^38^ has suggested that there is increased amount of chondrocyte deaths in the near regions of intra-articular ankle fractures. However, comparing our study to the study by Tochigi et al. could be problematic due to fracture not penetrating the joint cartilage in our subjects, whereas joint cartilage is macroscopically damaged in the study by Tochigi et al. As stated by Anderson et al.,^7^ the risk of developing a rapidly progressing post-traumatic OA seems to be highly related to the energy of the initial injury and cartilage surface damage. Another study, by Godoy-Santos et al.,^39^ displayed chondrocyte disarray in ankle cartilage shortly after unstable Weber type-B and -C fractures. It was also found that there was an increased collagen deposition in the synovial tissue, suggesting cartilage degradation via increased amounts of collagen being released to synovial fluid. Though in the study by Godoy-Santos et al. the cartilage samples were from the talar dome at the site of osteochondral lesion, unlike in our study, in which the cartilage surface is not visibly damaged. It should also be noted that previous studies have examined only talar cartilage,^38,39^ as we were able to detect differences in tibial cartilage. We may even speculate whether the cartilage is damaged at all in our subjects; the ECM of cartilage displays no clear degradation or lesions with T2 relaxation time. Whether chondrocytes are damaged is up to speculation: T2 relaxation time is not sensitive to chondrocytes due to the relatively low volume fraction of cells in cartilage.

It has been previously demonstrated that the severity of ankle injury is an important factor for developing post-traumatic ankle OA.^6,7^ However, we were not able to observe significant differences in cartilage ECM between stable and unstable ankle fractures. Therefore, we may speculate whether differences between the groups would be detected over a longer period after fracture, as the higher trauma energy seems to indicate faster post-traumatic cartilage degradation.^7^ Post-traumatic OA is thought to result from penetrating cartilage injuries, altered mechanical loading and chronic long-term inflammation due to tissue injury from trauma.^7,40,41^ Previous studies have demonstrated that inflammatory cytokines are significantly elevated already shortly after ankle fractures^39,42,43^ and remain elevated even after healing of the fracture.^44^ It could be that more extensive tissue damage results in more intense long-term inflammatory response, explaining why the differences in cartilage are not observed in the short-term between the fracture groups. However, specific inflammatory cytokines have not been able to predict development of post-traumatic OA yet.^45^ It should also be noted that the delay from injury to MRI varied between our patients due to practical challenges, such as MRI scanner accessibility. This variability could pose a potential source of error, as the T2 relaxation time could be influenced differently by physiological responses to injury during various stages of inflammation and healing process.

Previously, there have been only few studies assessing articular cartilage of the ankle joint with qMRI.^46–48^ Lee et al.^46^ observed lower T2 values with normal anterior talofibular ligament (ATFL) compared to partial or complete ATFL tear in patients with ankle pain. Wikstrom et al.^47^ displayed increase in T1ρ relaxation time with chronic ankle instability, whereas Krause et al.^48^ demonstrated higher T2* relaxation times in cavovarus patients with symptomatic ankle OA. It should be noted that none of these publications have evaluated the quality of articular cartilage shortly after trauma. Only few publications have assessed knee articular cartilage using qMRI shortly after anterior cruciate ligament (ACL) injuries.^49–53^ Similar to our findings, lower T2 values have been observed shortly after ACL injury by Casula et al.^49^ in the knee cartilage. Studies by Marchiori et al.^50^ and Zhong et al.^51^ describe T2 relaxation time of knee cartilage after trauma, before ACL repair, but do not specify the time between the trauma and qMRI measurements. Other groups have also assessed articular cartilage post-trauma using qMRI, but after a significantly longer period after injury.^52,53^

There are multiple strengths in our study. First, our study provides previously unreported association of stability of the ankle mortise, verified by valid clinical test, and T2 relaxation time. Second, the imaging of standard radiographs and MR of the isolated unilateral SER-type fractures were done shortly after injury, allowing us to evaluate the effect of the trauma without long-term effects of inflammation or altered mechanical function. Third, qMRI methods are non-invasive, allowing us to include total of 70 participants, which is considerably more than studies using tissue samples.^38,39,42^ Fourth, no previous studies have been performed to evaluate ankle cartilage shortly after injury using qMRI.

Our study has also some limitations. Though the cross-sectional nature of the study enables us to evaluate cartilage shortly after injury, it does not provide us with long-term data on how the quality of the cartilage develops over time. In the future, a longitudinal follow-up study using qMRI on the same subjects would provide information on how the cartilage is affected by the ankle fractures over time. Also, though we were able to include a total of 70 participants in this qMRI study, each studied group remained relatively small. The groups were not age- or BMI-matched, though this was accounted for in the statistical analysis and, furthermore, it does not explain our findings since lower age and BMI (of the control group) have been previously associated with lower T2 relaxation times.^27,33^ However, we believe that an age-matched control group should be included in future studies. We were also not able to analyze the cartilage in different zones due to thin ankle cartilage. For the same reason we decided not to include analysis of cartilage thickness. The synovial fluid resulting from trauma may slightly interfere with the analysis, as discussed previously; however, we consider it to result in only minor alterations to T2 relaxation times.

In conclusion, the severity of the ankle fracture, measured by MCS widening at external-rotation stress test, does not seem to increase cartilage ECM degradation immediately after trauma. Lower T2 relaxation times of tibial cartilage in fractured ankles suggest intact cartilage ECM. However, the clinical significance of T2 relaxation time in cartilage assessment shortly post-trauma is not completely clear and further studies are required to understand its potential and clinical applications.

## Supplemental Material

Supplemental Material - Assessment of articular cartilage of ankle joint in stable and unstable unilateral weber type-B/SER-type ankle fractures shortly after trauma using T2 relaxation time Click here for additional data file. Supplemental Material for Assessment of articular cartilage of ankle joint in stable and unstable unilateral weber type-B/SER-type ankle fractures shortly after trauma using T2 relaxation time by Sami Lehtovirta, Victor Casula, Marianne Haapea, Simo Nortunen, Sannamari Lepojärvi, Harri Pakarinen, Miika T. Nieminen, Eveliina Lammentausta, and Jaakko Niinimäki in Acta Radiologica Open
