# Supplementary material for: Assessment of articular cartilage of ankle joint in stable and unstable unilateral weber type-B/SER-type ankle fractures shortly after trauma using T2 relaxation time
Source: Acta Radiol Open. 2023 Sep 29;12(7):20584601231202033. doi: 10.1177/20584601231202033 (PMC10540593; doi:10.1177/20584601231202033)
Supplement: Supplemental Material - Assessment of articular cartilage of ankle joint in stable and unstable unilateral weber type-B/SER-type ankle fractures shortly after trauma using T2 relaxation time [file sj-pdf-1-arr-10.1177_20584601231202033.pdf]

SUPPLEMENTAL MATERIAL

Assessment of Articular Cartilage of Ankle Joint in Stable and Unstable Unilateral Weber Type-B/SER-type Ankle Fractures Shortly After Trauma Using T2 Relaxation Time

[Author names blinded for Review]

Cartilage segmentation and slice selection

T2 relaxation times were acquired from segmentation of sagittal T2 maps of ankle mortise to enable evaluation of tibial (TiC) and talar cartilage (TaC). One of the authors segmented the ankle cartilage using an in-house developed software programmed with Matlab (MATLAB, Mathworks Inc., Natick, MA). The segmentation was conducted twice for each ankle to ensure utmost quality; the second segmentation was considered definitive. The anterior and posterior edge of tibial cartilage were considered as the anterior (AE) and posterior end (PE), respectively, for TiC and TaC. The cartilage between AE and PE was divided into three regions of equal width for both TiC and TaC. These three areas were named as anterior region (AR), middle region (MiR) and posterior region (PR) (Fig. 1). We also wanted to avoid partial volume averaging; therefore, we did not include any of the surfaces with significant inclination (such as medial or lateral malleolar surfaces). Also, high intensity pixels in the synovium located between TiC and TaC surfaces were excluded if the T2 relaxation time was higher than 90 milliseconds (ms) or if the pixel was clearly not located within cartilage bounds. This was done to avoid inclusion of synovial fluid.

After segmentation, a total of six (6) slices from each ankle was chosen: two slices from each section (medial, central, lateral) (Fig. 2). The two slices from each section were combined as one with size-weighted averages to represent medial, central, and lateral sections. If the most medial or lateral slice displayed inclination of the articular cartilage surface, the slice was omitted from the analysis resulting in a single slice representing that section. Also, if an ankle was too narrow that six slices could not be extracted, the middle slice would represent the central section. The three sections

(medial, central, lateral) and the three regions (AR, MiR, PR) of these sections resulted in nine (9) regions of interest (ROIs) for both TiC and TaC (total of 18 ROIs).
